# Expression analysis of Rab11 during zebrafish embryonic development

**DOI:** 10.1186/s12861-019-0207-7

**Published:** 2019-12-30

**Authors:** Haijun Zhang, Yu Gao, Peipei Qian, Zhangji Dong, Wenjin Hao, Dong Liu, Xuchu Duan

**Affiliations:** 1 Co-innovation Center of Neuroregeneration, Jiangsu Key Laboratory of Neuroregeneration, Nantong University, 19# Qixiu Road, Nantong, 226001 China; 2 College of life science, Nantong University, 9# Seyuan Road, Nantong, 226001 China

**Keywords:** *rab11*, Zebrafish, Expression

## Abstract

**Background:**

Rab proteins are GTPases responsible for intracellular vesicular trafficking regulation. Rab11 proteins, members of the Rab GTPase family, are known to regulate vesicular recycling during embryonic development. In zebrafish, there are 3 *rab11* paralogues, known as *rab11a*, *rab11ba* and *rab11bb*, sharing high identity with each other. However, the expression analysis of *rab11 is* so far lacking.

**Results:**

Here, by phylogeny analysis, we found the three *rab11* genes are highly conserved especially for their GTPase domains. We examined the expression patterns of *rab11a*, *rab11ba* and *rab11bb* using RT-PCR and in situ hybridization. We found that all the three genes were highly enriched in the central nervous system, but in different areas of the brain. Apart from brain, *rab11a* was also expressed in caudal vein, pronephric duct, proctodeum, pharyngeal arches and digestive duct, *rab11ba* was detected to express in muscle, and *rab11bb* was expressed in kidney, fin and spinal cord. Different from *rab11a* and *rab11ba*, which both have maternal expressions in embryos, *rab11bb* only expresses during 24hpf to 96hpf.

**Conclusions:**

Our results suggest that *rab11* genes play important but distinct roles in the development of the nervous system in zebrafish. The findings could provide new evidences for better understanding the functions of rab11 in the development of zebrafish embryos.

## Background

Rab/Ypt proteins are small GTP-binding proteins of the Ras superfamily and are key players in intracellular vesicular trafficking regulation [1, 2]. RAB11 was first found from cultured MDCK cell cDNA library screening for homologous genes of yeast YPT1/SEC4 [3], and later identified as the 11th member of the Rab family [4]. Among various Rab proteins, Rab11 is known to localize to recycling endosome and regulate vesicular recycling and primary ciliogenesis, playing a key role in Kupffer vesicle development in zebrafish [5–7].

The Rab11 family is composed of three members of GTPases, Rab11a, Rab11b and Rab25, based on specific motifs [8]. Murine Rab11a and Rab11b were found to localize in distinct cellular compartment, suggesting functional differentiation which have been further proved by several recent studies [9]. For instances, Rab11b but rather Rab11a was found to be responsible for endosomal recycling of fibroblast growth factor receptor 4 (FGFR4), a signaling receptor for maintaining tissue homeostasis [10]. In Hela cells, Rab11b but not Rab11a is essentially required for recycling of protease-activated receptor-1 (PAR1), however, depletion of Rab11a could disrupt lysosomal degradation of PAR1 and therefore enhance its intracellular accumulation [11]. Haugsten et al. have just reported that both Rab11a and Rab11b contribute to maintain the endosomal-lysosomal pathway where Rab11a plays the major roles [12]. Although Rab11a subcellular localization has been demonstrated by transgenic zebrafish [13], and molecular function of Rab11 proteins in primary cilia membrane assembly in Kupffer Vesicle has been studied [7], the developmental expression profile of Rab11 proteins in zebrafish remains elusive. In the present project, we showed that zebrafish *rab11* genes are conserved in vertebrate evolution by *in silicon* analysis. We then analyzed the temporal and spatial expression of *rab11* genes in embryonic development by RT-PCR and whole mount in situ hybridization. Our study provides new insight into the *rab11* expression and promotes the use of this model organism to tackle future studies on the role of *rab11* in embryo development.

## Results

### *rab11* genes are highly conserved in vertebrates

We first examined Rab11 protein phylogeny in zebrafish [Rab-11A (NP_001007360); Rab-11BA (NP_999935); Rab-11BB (NP_001002555)] and other representative organisms including worm [*Caenorhabditis elegans*, RAB-11.1 (NP_490675); RAB-11.2 (NP_001251691)], fruit fly [*Drosophila melanogaster*, Rab11 isoform A (NP_599137); Rab11 isoform B (NP_477170)], medaka [*Oryzias latipes*, Rab-11A (XP_004086096); Rab-11B (XP_004068250); Rab-11B-like (XP_004068248)], frog [*Xenopus tropicalis*, Rab-11A (NP_001016481); Rab-11B.1 (NP_001120303)], chicken [*Gallus gallus*, Rab-11A (NP_001005827); Rab-11B (NP_001012569)], mouse [*Mus musculus*, Rab-11A (NP_059078); Rab-11B (NP_033023)], rat [*Rattus norvegicus*, Rab-11A (NP_112414); Rab-11B (NP_116006)] and human [*Homo sapiens*, Rab-11A isoform 1 (NP_004654); Rab-11B (NP_004209)].

**Fig. 1:**
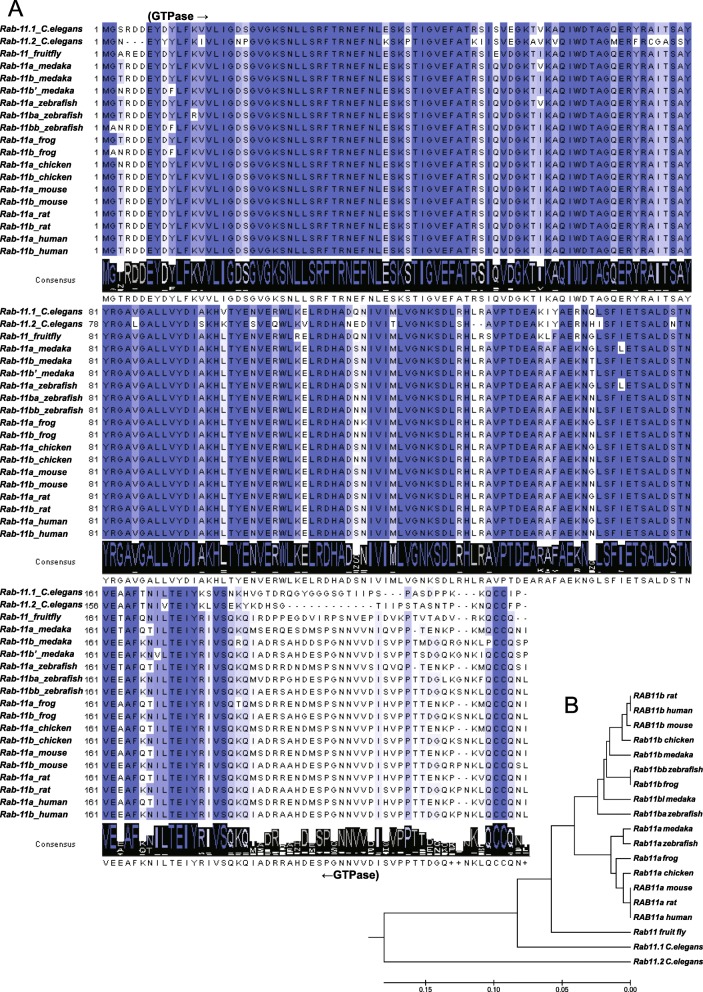
Rab11 is highly conserved during evolution. **a** Alignment of Rab11 protein amino acid residue sequences of *Caenorhabditis elegans*, *Danio rerio*, *Oryzias latipes*, Xenopus tropicalis, *Gallus gallus*, *Mus musculus*, *Rattus norvegicus* and *Homo sapiens*. The sequences accessions IDs are listed following respectively, NP_490675, NP_001251691, NP_599137, NP_477170, XP_004086096, XP_004068250, XP_004068248, NP_001016481, NP_001120303, NP_001005827, NP_001012569, NP_059078, NP_033023, NP_112414, NP_116006, NP_004654, NP_004209. These protein sequences were aligned using ClustalW2 program and edited by Jalview. The GTPase domain sequence is indicated. **b** Phylogenetic tree of amino acid sequences generated using the MEGA6 software

The zebrafish Rab11a, Rab11ba and Rab11bb proteins are highly conserved during evolution, especially the GTPase domain, suggesting their important functions. While these paralogues differ in the C-terminal part adjacent to the GTPase domain (Fig. 1a). Zebrafish *rab11a* was clustered in the *rab11a* group, and *rab11ba* and *rab11bb* in the *Rab11b* group (Fig. 1b) in the phylogenic tree constructed according to amino acid sequences of all the Rab11 proteins above.

### Expression of *rab11* genes analyzed by RT-PCR

**Fig. 2:**
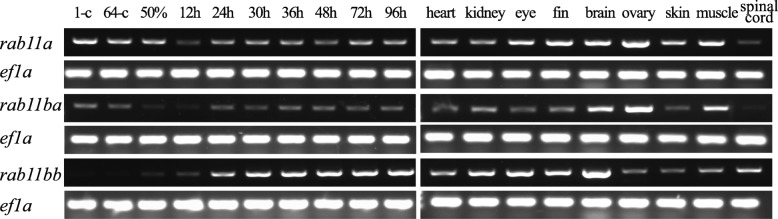
Expression of *rab11* genes analyzed by RT-PCR. RT-PCR analysis of *rab11a*, *rab11ba* and *rab11bb* expression in embryonic zebrafish at different stages and adult tissues and organs

We examined *rab11a* expression using RT-PCR. High expression level of maternal *rab11a* was detected in both 1-cell stage fertilized eggs and cleavage stages (Fig. 2). *Rab11a* exhibited steady expression through 96 hpf with a slight lower level found at 12 hpf. In adult zebrafish, *rab11a* was found highly expressed in the eye, fin, brain, eggs and muscle. Its expression in the nervous system is much restricted to the brain, being very weak in the spinal cord. With much similarity to *rab11a*, *rab11ba* is steadily expressed from 1-cell stage through 96 hpf, except for a slightly low expression period around 12 hpf. Like *rab11a*, *rab11ba* is maternally expressed, as detected at 1-cell stage and in the eggs. *Rab11ba* is also highly expressed in the brain, eggs and muscle and is very weak in the spinal cord. As a paralogue, *rab11bb* was expressed differently. *Rab11bb* only showed extremely weak maternal mRNA expression. High zygotic *rab11bb* expression was not observed until 24 hpf and remained through 96 hpf. *Rab11bb* was detected at high level in kidney, eye, fin and brain, and weakly expressed in muscle. Unlike *rab11a* and *rab11ba*, *rab11bb* expression level in the spinal cord is high.

### *Rab11a* expression during zebrafish embryonic development

**Fig. 3:**
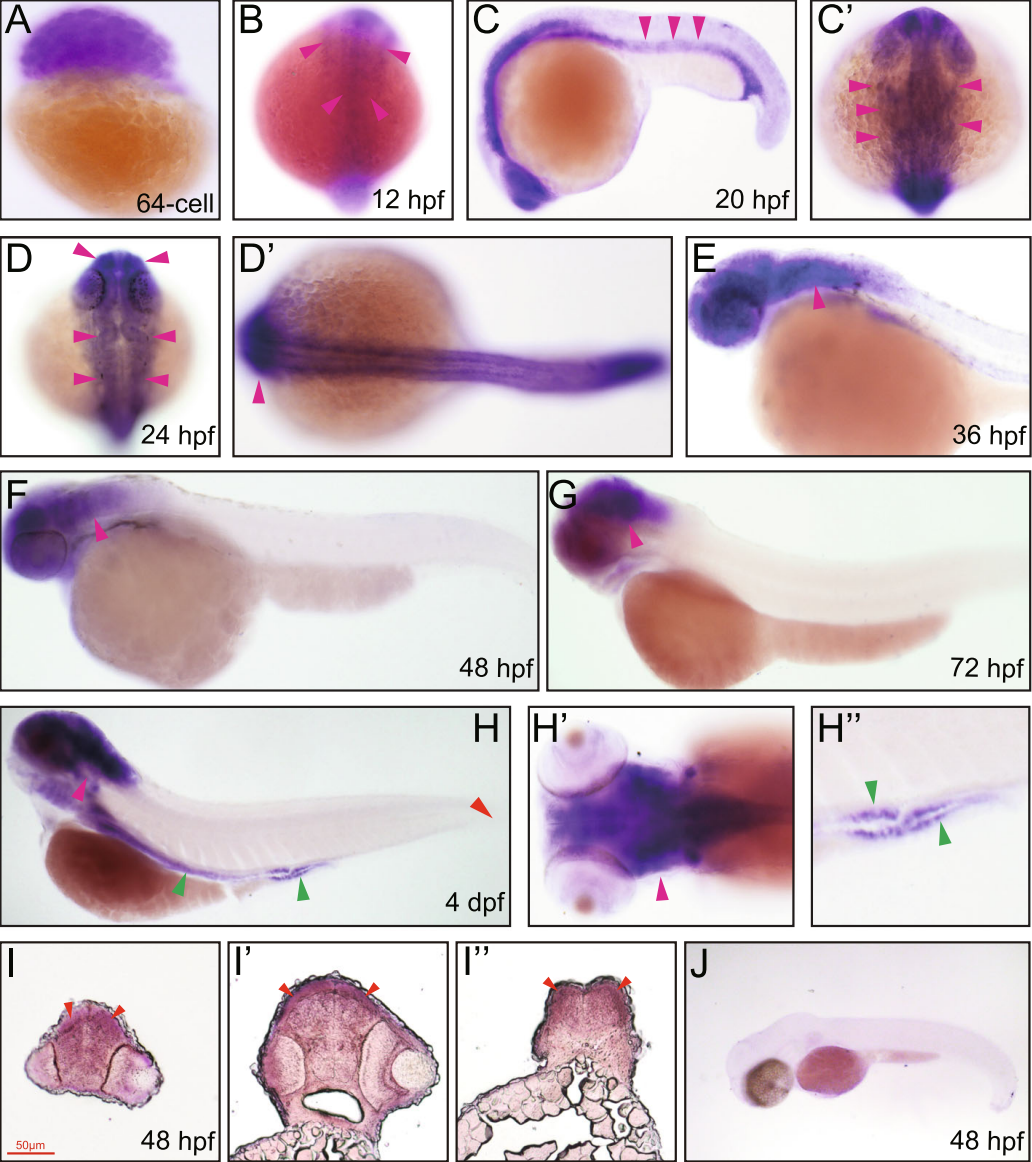
Whole mount in situ and transverse section hybridization analysis of *rab11a* in zebrafish embryos. **A** 64 cell, lateral view. **B** 12 hpf, dorsal view, red arrowheads indicate the ubiquitous expression. **C** 20 hpf, lateral view, red arrowheads indicate endoderm. **C’** 20 hpf, dorsal view, red arrowheads indicate brain. **D** 24 hpf, dorsal view, red arrowheads indicate brain. **D’** 24 hpf, dorsal view, red arrowheads indicate hindbrain. **E** 36 hpf, lateral view, red arrowhead indicates brain. **F** 48 hpf, lateral view, red arrowhead indicates brain. **G** 72 hpf, lateral view, red arrowhead indicates brain. **H** 4 dpf, lateral view, red arrowhead indicates brain; green arrowheads indicate pronephric duct. **H’** 4 dpf, dorsal view, arrowhead indicates brain. **H”** 4 dpf, lateral view, green arrowheads indicate pronephric duct. **I** 48 hpf, the trunk transverse section of the embryonic forebrain, arrowheads indicate cerebral cortex. **I’** 48 hpf, the trunk transverse section of the embryonic midbrain, arrowheads indicate cerebral cortex. **I”** 48 hpf, the trunk transverse section of the embryonic hindbrain, arrowheads indicate cerebral cortex. **J** 48 hpf, lateral view, embryos stained with the sense *rab11a* probe

**Fig. 4:**
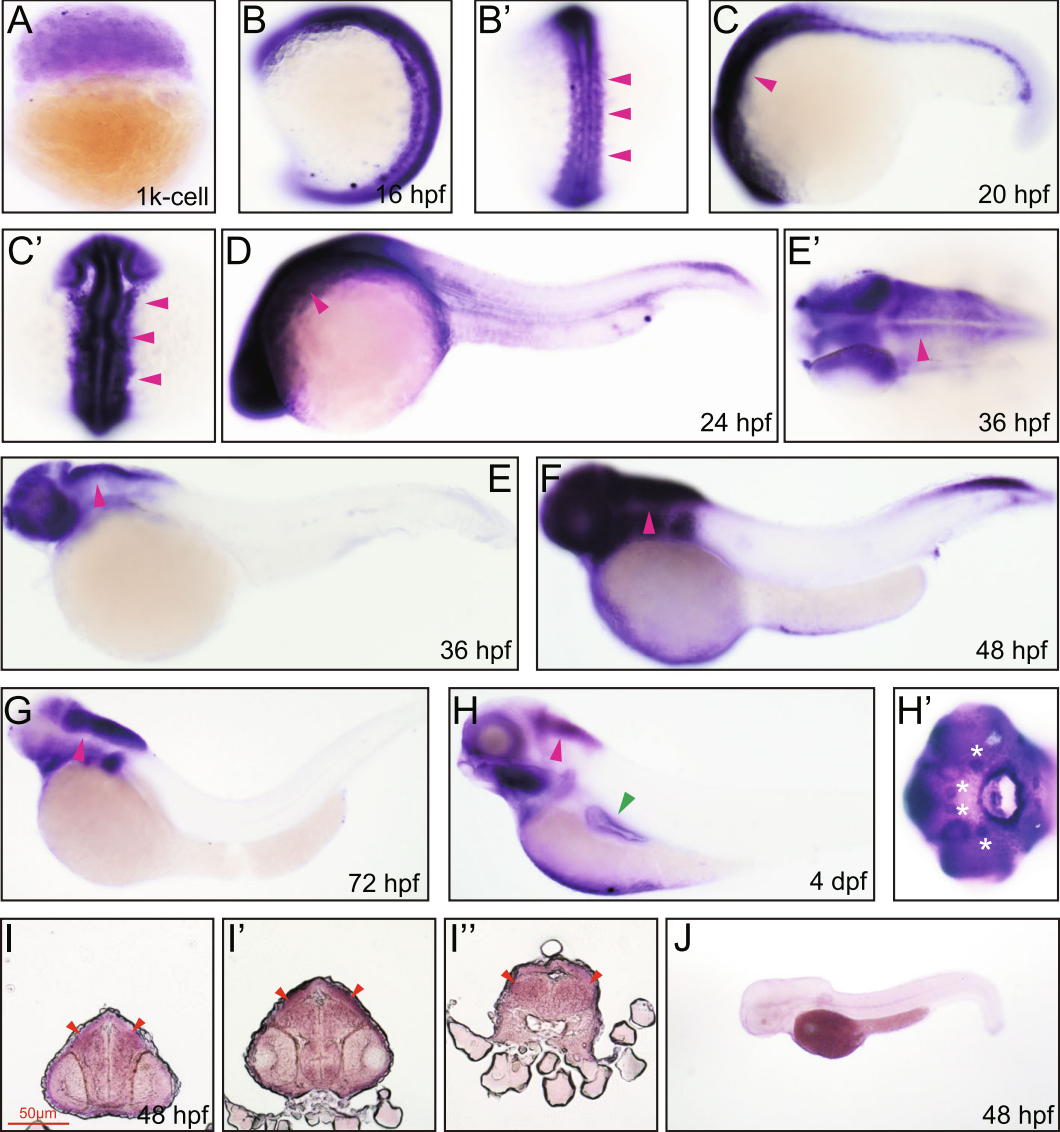
Whole mount in situ and transverse section hybridization analysis of *rab11ba* in zebrafish embryos. **A** 1 k cell, lateral view. **B** 16 hpf, lateral view. **B’** 16 hpf, dorsal view, arrowheads indicate neural tube. **C** 20 hpf, lateral view, arrowhead indicates brain. **C’** 20 hpf, dorsal view, arrowheads indicate brain. **D** 24 hpf, lateral view, arrowhead indicate brain. **E** 36 hpf, lateral view, arrowhead indicates spinal cord. **E’** 36 hpf, dorsal view, arrowhead indicates brain and head structure. **F** 48 hpf, lateral view, arrowhead indicates spinal cord. **G** 72 hpf, lateral view, arrowhead indicates brain. **H** 4 dpf, lateral view, red arrowhead indicates brain, green arrowhead indicates pectoral fins. **H’** 4 dpf, asterisks indicate neuromasts. **I** 48 hpf, the trunk transverse section of the embryonic forebrain, arrowheads indicate cerebral cortex. **I’** 48 hpf, the trunk transverse section of the embryonic midbrain, arrowheads indicate cerebral cortex. **I”** 48 hpf, the trunk transverse section of the embryonic hindbrain, arrowheads indicate cerebral cortex. **J** 48 hpf, lateral view, embryos stained with the sense *rab11ba* probe

**Fig. 5:**
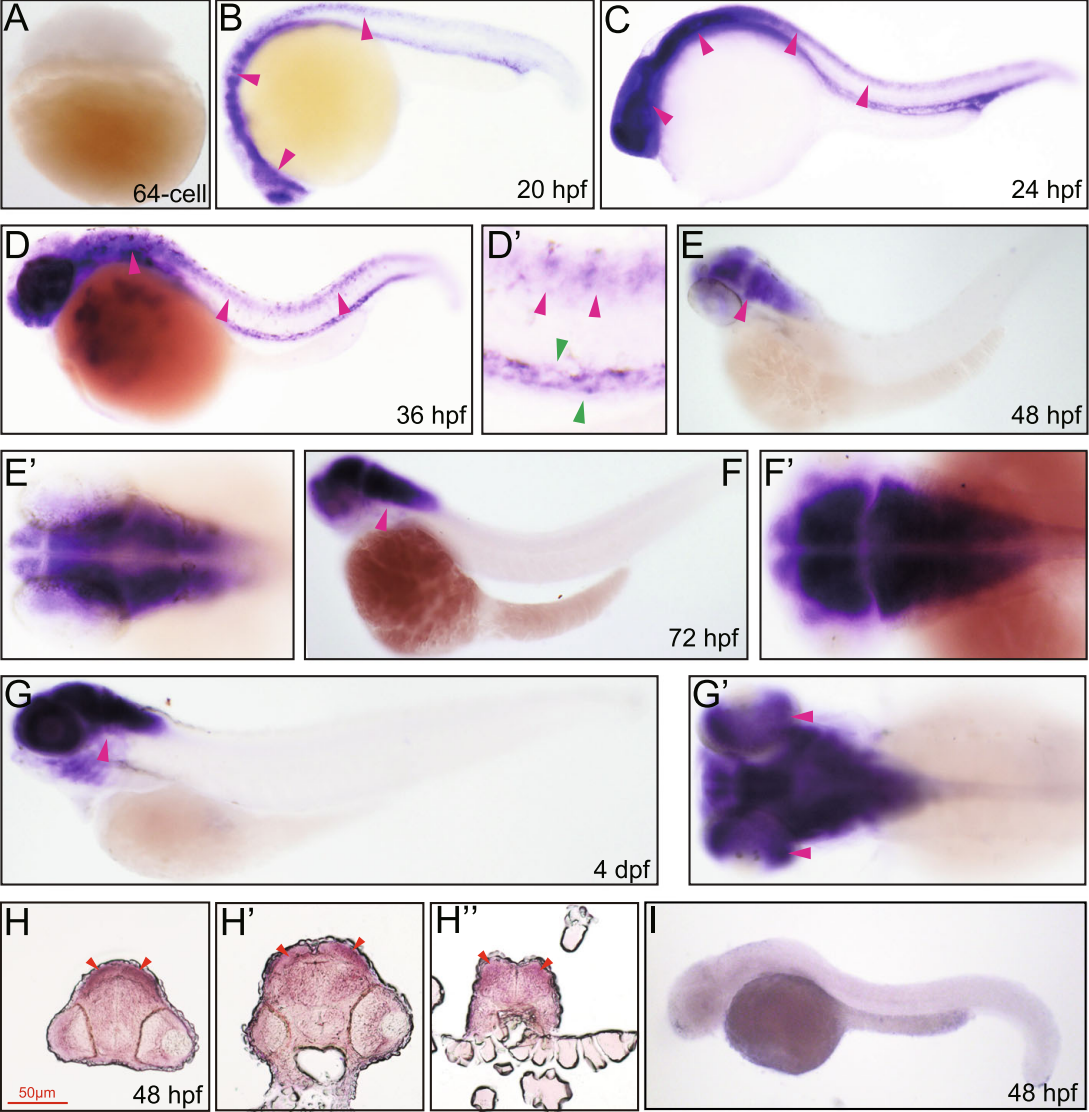
Whole mount in situ and transverse section hybridization analysis of *rab11bb* in zebrafish embryos. **A** 64 cell, lateral view. **B** 20 hpf, lateral view, arrowheads indicate brain and spinal cord. **C** 24 hpf, lateral view, arrowheads indicate brain and spinal cord. **D** 36 hpf, lateral view, arrowheads indicate spinal cord. **D’** 36 hpf, dorsal view, red arrowheads indicate spinal cord, green arrowheads indicate pronephric duct. **E** 48 hpf, lateral view, arrowhead indicates brain. **E’** 48 hpf, dorsal view. **F** 72 hpf, lateral view, arrowhead indicates ear. **F’** 72 hpf, dorsal view. **G** 4 dpf, lateral view, arrowhead indicates pharyngeal arches. **G’** 96 hpf, dorsal view, arrowhead indicates retina. **H** 48 hpf, the trunk transverse section of the embryonic forebrain, arrowheads indicate cerebral cortex. **H’** 48 hpf, the trunk transverse section of the embryonic midbrain, arrowheads indicate cerebral cortex. **H”** 48 hpf, the trunk transverse section of the embryonic hindbrain, arrowheads indicate cerebral cortex. **I** 48 hpf, lateral view, embryos stained with the sense *rab11bb* probe

We then performed in situ hybridization to further study the spatial-temporal expression pattern of *rab11a* as well as *rab11ba* and *rab11bb* (Figs. 3, 4, 5). High level of maternal *rab11a* mRNA was detected at 2 hpf (64-cell) (Fig. 3A). At 12 hpf, zygotic *rab11a* mRNA was found in the whole organism without tissue specificity (Fig. 3B). As seen at 20 hpf to 24 hpf, *rab11a* expression began to decrease in the trunk and was mainly restricted to the brain and head structures, and also in the caudal spinal cord, caudal vein, pronephric duct and the proctodeum (Fig. 3C-D’). The mRNA expression in the brain remained strong until 36 hpf and decreased in other tissues (Fig. 3E). From 48 hpf to 72 hpf, *rab11a* mRNA was expressed mainly in the brain, with expression in the posterior hindbrain becoming weaker (Fig. 3F, G). At 48 hpf, *rab11a* expression was also confirmed to express in the brain especially the cerebral cortex by histological analysis of sectioned embryos (Fig. 3I-I”). At 4 dpf, *rab11a* was strongly expressed in the brain and also in the pharyngeal arches and the digestive duct (Fig. 3H-H”).

### *Rab11ba* expression during zebrafish embryonic development

Similar to *rab11a*, maternal *rab11ba* mRNA was detected at 3 hpf (1 k-cell) (Fig. 4A). At 16 hpf, *rab11ba* was expressed in the whole organism and especially in the neural tube (Fig. 4B-B’). At 20 hpf, *rab11ba* expression in the brain remained strong, but its mRNA was not detected in the spinal cord (Fig. 4C, C’). Like that of *rab11a*, *rab11ba* mRNA was seen in the pronephric duct and proctodeum (Fig. 4C). At 24 hpf, *rab11ba* was maintained in the brain (Fig. 4D). At this stage, *rab11ba* started its expression in the pectoral fins. *Rab11ba* expression in the spinal cord and the caudal vein was significantly decreased at 36 hpf and again increased at 48 hpf (Fig. 4E-F). At this stage, *rab11ba* was expressed at proctodeum at 48 hpf. Transverse section analysis demonstrated almost the same expression patterns of *Rab11ba* as that of *Rab11a* where it was expressed in the brain at 48 hpf as well (Fig. 4I-I”). From 72 hpf to 96 hpf, *rab11ba* was expressed in the brain, retina, olfactory placode and pectoral fins (Fig. 4G-H’). Rab11ba was also found highly expressed in cranio-facial neuromasts at 72 hpf and 96 hpf, which was unique compared to rab11a and rab11bb (Fig. 4G-H’).

### *Rab11bb* expression during zebrafish embryonic development

No maternal message was detected of *rab11bb* at 2 hpf (64-cell) (Fig. 5A). *Rab11bb* expression was seen in the brain, retina, and spinal cord except for the caudal part and the proctodeum from 20 hpf to 24 hpf (Fig. 5B, C). Its expression in the spinal cord turned weaker at 36 hpf (Fig. 5D, D’). At 48 hpf and 72 hpf, *rab11bb* expression was restricted to the brain, with expression in the retina and ear weakly detected and no detectable expression in other tissues (Fig. 5E-F’). Similar to *rab11a* and *rab11ba*, *rab11bb* was also observed to express in the brain at 48 hpf but with a seemingly lower level by comparing the hybridization signals (Fig. 5H-H”). At 4 dpf, its expression in the brain remained strong, while its mRNA was also detected in the retina, ear and pharyngeal arches (Fig. 5G, G’).

## Discussion

Here we described the developmental expression patterns of *rab11a*, *rab11ba* and *rab11bb*. So far, although it has been reported that *rab11a* is expressed in epithelia [14] and myoblasts [15] in fruit fly, and in various tissues in mouse and human [16], the developmental expression of these genes in a whole organism was not described. It was also reported that Rab11a and Rab11b were localized to distinct cellular compartments in mouse [9]. The different expression patterns indicate the *rab11* genes play diverse roles during development. In recent years, studies have proved the functional relationships between rab11 family proteins and neuron system development [17]. For instances, rab11 was reported to be a regulator of presynaptic function and axon regeneration [18, 19], Rab11-dependent recycling pathway was revealed to be involved in regulating cortical neuronal migration via the regulation of N-cadherin trafficking [20, 21]. in *Drosophila*, rab11 was found to regulate dendrite pruning of sensory neurons via regulating degradation of Neuroglian [22].

In this study, we found all the three *rab11* paralogues were expressed in the brain from 24 hpf to 72 hpf, suggesting their essential function during brain development and neural development. However, the three rab11 genes also showed differences in their expression patterns which implied distinct functions of them. For, example, we found rab11ba rather than the other two rab genes was highly expressed in cranio-facial neuromasts. In neuromasts, hair cells sense stimuli from the outside with their apical cilia, whose development and function are regulated by rab11ba [7]. Therefore, rab11ba might be essential for the development and function of hair cells in zebrafish. Besides, rab11a and rab11ba were also found to express in caudal vein at around 24 hpf, suggesting their potential regulating roles in vascular development of zebrafish embryos. More clues to the functional differentiation of the three rab genes need to be obtained in future studies.

## Conclusions

There are three rab11 genes named *rab11a*, *rab11ba* and *rab11bb* in zebrafish. In this study, we performed the phylogenetic analysis of these Rab11 proteins of zebrafish and found they are highly conserved among diverse animal species. Further investigations using RT-PCR and in situ hybridization demonstrated the expression patterns of *rab11* genes during zebrafish embryonic development. The results indicated that these rab11 genes might play vital roles during nervous system development. These findings could provide new evidences for better understanding the functions of rab11 in the development of zebrafish embryos.

## Methods

### Zebrafish tissue and embryos

The zebrafish (AB line) in this study was obtained from China Zebrafish Resource Centre and maintained in Jiangsu key laboratory of neuroregeneration of Nantong university. Animal experiments were conducted conforming to the local institutional laws and the Chinese law for the Protection of Animals. Embryos were obtained through natural mating (AB line) and maintained at 28.5 °C. Embryonic stages are as described [23]. Embryos after 24 hpf were treated with 0.2 mM 1-phenyl-2-thio-urea (PTU). Zebrafish embryos were collected at various stages, fixed with 4% paraformaldehyde (PFA) in phosphate-buffered saline (PBS) overnight at 4 °C or 2 h at room temperature, washed with PBST, dehydrated in methanol and stored at − 20 °C until use. Embryos younger than 24 hpf were dechorionated after fixation, prior to storage.

### Bioinformatics

The zebrafish rab11a, rab11ba and rab11bb sequence and conserved domains information was got form Ensembl (rab11a: ENSDARG00000041450, rab11ba: ENSDARG00000041878, rab11bb: ENSDARG00000090086). Rab11 sequences were aligned by the ClustalW2 program (http://www.ebi.ac.uk/Tools/msa/clustalw2/). And the phylogenetic tree was built by MEGA6 software. Primers for PCR were designed by the Primer Premier 5 software.

### RNA extraction, reverse transcription, and RT-PCR

Tissue was homogenized and frozen in TRIzol Reagent (Invitrogen) and stored at − 80 °C. The RNA was extracted following the manufacturer’s instruction. 1 μg of RNA was reverse transcribed into cDNA by the use of Transcriptor First Strand cDNA Synthesis Kit (Roche) according to the manufacturer’s instructions. Synthesized cDNA was stored at − 20 °C. The primers for RT-PCR are listed, for *ef1a*: left primer ef1a-1-L TGATCTACAAATGCGGTGGA, right primer ef1a-1-R CAATGGTGATACCACGCTCA (141 bp); for *rab11a*: left primer, AAGCCGTTAGCATGGCTACT; right primer, CTTTTATTGCCCACCAGCAT (502 bp); for *rab11ba*: left primer, CAGGACAGGAACGCTACAGA; right primer, CACAACACACAGGAGCGAAA (540 bp); for *rab11bb*: left primer, CTGCTGTCGAGATTCACACG; Right primer, CGTGGTTACAGGTTTTGGCA, (584 bp). All PCR amplifications were carried out in a total volume of 50 μl using specific primers and Advantage2 Polymerase Kit (Clontech).

### Whole mount in situ hybridization and sectioned histological analysis

*Rab11a*, *rab11ba* and *rab11bb* cDNA fragments were cloned into pGEM-T easy vector (Promega) with primers described in Section 2.3. RNA extraction, reverse transcription, and RT-PCR. Digoxigenin (DIG)-labeled RNA sense and antisense probes were made from the linearized plasmids according to the manufacturer’s protocol using the DIG RNA Labeling Kit (SP6/T7) (Roche). The procedure was modified from Thisse in situ hybridization protocol [24]. The small baskets were not used in our protocol. BM purple AP substrate (Roche) was used instead of the staining solution. We use the BBR (Boehringer blocking reagent, Roche) for blocking.

For sectioned histological analysis, the whole-mount in situ hybridized embryos were fixed in 4% PFA for 2 h at room temperature and then washed with PBST three times for 5 min each to remove the fixative reagents. The procedure was followed by a two-step embedment: one with 1.5% agarose–5% sucrose solution for adjusting the fish orientation, and the other with Tissue-Tek OCT compound (SAKURA). The embryos were then sectioned with a Leica RM2125 microtome at 12 μm. After drying for 4 h at 37 °C, the sectioned samples were washed again with PBS and mounted and coverslipped with mounting medium. Pictures were taken with an Olympus DP70 camera on an Olympus stereomicroscope MVX10.

## Data Availability

All data generated and analyzed during this study are included in this published acticle.

## Abbreviations

BBR: Boehringer blocking reagent
DIG: Digoxigenin
dpf: Days post fertilization
hpf: Hours post fertilization
PBS: Phosphate-buffered saline
PBST: Phosphate-buffered saline tween-20
PFA: Paraformaldehyde
PTU: 1-phenyl-2-thio-urea
RT-PCR: Reverse transcription polymerase chain reaction
